# Tea Drinking

**Published:** 1857-10

**Authors:** 


### TEA DRINKING.

If the question be narrowed down to "Tea, or no Tea," we advocate the weed. The world will be the happier and healthier by the moderate use of any of the China teas, in their purity, than without them. The immoderate use of cold water is prejudicial to health, whether as a drink or a lavement, and so is the immoderate use of bread and butter. It is the argument of a fanatic to say, that because the excessive use of anything is injurious, it should, therefore, be discarded altogether.

Chemistry decides that the essential elements of coffee and tea are identical, and are nutritious.

Tea is a stimulant, and so is any other nutritive article. That which imparts no stimulus is not fit for food. An ordinary meal stimulates the pulse to a greater activity by 5 or 10 per cent.

Tea, being used warm, and at meal time, promotes digestion by its warmth, as any other warm drink would do.

Any cold drink, even water, taken at meal time, arrests the progress of digestion, until it is raised to a heat of about a hundred degrees, and if that arrest be too long protracted, convulsions follow, and sometimes death—as has happened to children many times by eating a couple of hard-boiled eggs hastily, or upon an empty stomach, or, indeed, eating much of any indigestible article.

Thus it is that, as far as the use of tea at our meals banishes the use of cold water at meals, it is a safeguard.

Late and hearty suppers destroy multitudes, either outright in a night, or in the insidious progress of months and years. It is almost the universal custom to take tea for supper. It is a stimulant. It aids the stomach in digesting more than it would have done, just in proportion to its stimulating qualities. And as all eat too much at supper time, the general use of warm tea as a drink at the last meal of the day is beneficial in the direction just named.

True wisdom lies in the moderate use of all the good things of this life.

It is stated, that at a tea party of sixty old women in England, it was ascertained that they were the mothers of eight hundred and sixty-nine children.

The presumption is, that these women were tea drinkers habitually, and it is equally inferrible that they did not drink it very "weak;" yet they were healthy enough to be old, and healthy enough to be the mothers of large families. An isolated fact proves nothing, but this one is suggestive.

It is then safer and healthier to take a cup of warm tea for supper than a glass of cold water.

With our habits of hearty suppers, it is better to take a cup of warm tea than to take no drink at all.

By the extravagant use of tea, many persons pass their nights in restlessness and dreams, without being aware of the cause of it. We advise such to experiment on themselves, and omit the tea altogether at supper, for a few times, and notice the result.

If you sleep better, it is clear that you have been using too much tea, in quantity or strength.

In order to be definite, we consider the following to be a moderate use of tea: a single cup at each meal as to quantity; as to strength, measure it thus: put a teaspoonful in a hot teapot; pour on a quart of boiling water; two-thirds of a teacup of this, adding a third of cream, or boiling milk, or hot water, with sugar or not: this is strong enough.

We believe that such use of China teas, by excluding cold drinks at our meals, and by their nutritious and pleasantly stimulating character, may be practiced for a lifetime, to very great advantage, without any drawback whatever; coffee also.

We believe that the world, and all that is created upon it, is for man, and that the rational use of its good things will promote the health and happiness of all mankind.

